# *Artemisia annua* L.: evidence of sesquiterpene lactones’ fraction antinociceptive activity

**DOI:** 10.1186/1472-6882-14-266

**Published:** 2014-07-28

**Authors:** Fabricio de Faveri Favero, Rogério Grando, Fabiana R Nonato, Ilza MO Sousa, Núbia CA Queiroz, Giovanna B Longato, Rafael RT Zafred, João E Carvalho, Humberto M Spindola, Mary A Foglio

**Affiliations:** CPQBA, University of Campinas, P.O. Box 6171, 13083-970 Campinas, SP Brazil; Department of Pharmacology, Anesthesiology and Therapeutics, Faculty of Dentistry, University of Campinas, P.O. Box 52, 13414-903 Piracicaba, SP Brazil

## Abstract

**Background:**

*Artemisia annua* L. has been used for many centuries in Chinese traditional medicine. Artemisinin, the active principle was first isolated and identified in the 1970s becoming the global back bone to the fight against malaria. Our research group previously developed an economic and ecological friendly process to obtain this compound. In the pursuit to also exploit the residue generated throughout the process we further evaluated the pharmacological potential of that extract.

**Methods:**

The alcoholic crude extract after artemisinin precipitation maintained an enriched sesquiterpene lactones content with residue artemisinin (1.72%) and deoxyartemisinin (0.31%), used as chemical markers for this sample. This study evaluated the pharmacological potential of the enriched sesquiterpene lactone fraction (Lac-FR) on different nociceptive and inflammatory experimental animal models. Previous findings on the biological properties of lactones obtained from natural products permitted us to explore the antinociceptive activities of these compounds based on *in vivo* chemical-induced behavioral assays.

**Results:**

The enriched sesquiterpene lactone fraction (Lac-FR) was administrated by intraperitoneal injection producing a relevant reduction in the reaction time of the animals in both phases of the formalin test, significantly reduced the sensitivity to mechanical allodynia stimulus, reduced the paw edema caused by carrageenan injection and promoted high antinociceptive activity in tail flick model suggesting relationship with the opioid system. Further studies are being undertaken to elucidate the active components involved with the antinociceptive activity as well as evaluation of synergy effect that is seen by combination of substances that is greater than would be expected from consideration of individual contributions.

**Conclusion:**

For the first time, results presented herein provided consistent data to support the potential use of these lactones for pain relief as revealed by chemical-induced nociception assays in mice.

## Background

There has been a substantial amount of research into the use of plants in folk-medicine, which typically highlight the importance of members of the Asteraceae family [1]. The genus *Artemisia* comprises over 400 species, many of which have an aromatic, bitter taste [2]. The herb *Artemisia annua* L. has been used for many centuries in Chinese traditional medicine as a treatment for fever and malaria [3, 4].

Sesquiterpene lactones (SLs) are a group of secondary metabolites found across the plant kingdom comprising a large group of over 5000 known compounds, being most common in families such as Cactaceae, Solanaceae, Araceae, and the Euphorbiaceae. However they are most prevalent in the Asteraceae, where they can be found ubiquitously [1, 5].

According to Mefort (2011) [6] sesquiterpene lactones are the most prevalent and biologically significant secondary metabolites since they are involved in a wide array of biological activities. Their structural diversity and variety of biological activities such as anticancer, anti-inflammatory, anti-tumor, anti-malarial, antiviral, antibacterial, antifungal *etc*. have prompted interest among chemists towards drug discovery research [1, 5].

In 1971, Chinese chemists identified artemisinin, a sesquiterpene lactone, isolated from leaves of *Artemisa annua* L., responsible for antimalarial activity, also denominated qinghaosu (QHS, artemisinin). This compound bears a peroxide group that unlike most other antimalarials, lacks a nitrogen-containing heterocyclic ring system [3].

Among the sesquiterpene lactones identified from the plant species are artemisinin, arteannuin B (two to four times) and artemisininic acid (seven to eight times) [2].

In order to exploit the by-product generated in the process of atemisinin extraction from *A. annua* L. our research group was prompted to study pharmacological activities of that material. Artemisinin, dihydro-epideoxyarteannuin-B and deoxyartemisinin were isolated from the sequiterpene lactone-enriched fraction obtained from the crude ethanolic extract of *A. annua* L. These compounds were tested on ethanol and indomethacin-induced ulcer models. Artemisinin did not afford cytoprotection under the experimental models tested. Only dihydro-epideoxyarteannuin- B and deoxyartemisinin decreased the ulcerative lesion index produced by ethanol and indomethacin in rats. These compounds did not demonstrate antiulcerogenic activity when tested on the ethanol-induced ulcer model, with previous administration of indomethacin, suggesting that the antiulcerogenic activity was a consequence of prostaglandin synthesis increase [7–10].

According to Chaturvedi (2011) [5] although, the exact mechanism of action of sesquiterpene lactones are not well understood they have been documented. Some authors have reported explanations to account for the role that those compounds play in anti-inflammatory activity in different experimental models evaluated [1, 11].

Based on previous work reported on anti-inflammatory activity of sesquiterpene lactones we were prompted to further study the antinociceptive and anti-inflammatory activity of the enriched sesquiterpene lactone fraction generated in the production of artemisinin from *A. annua* L.

## Methods

### Phytochemistry

#### Material plant and fractionation

*Artemisia annua* L. leaves (hybrid CPQBA2/39 x PL5) were collected from the experimental field of CPQBA/UNICAMP. Voucher specimen is deposited at CPQBA/UNICAMP under registration number 229. The hybrid varieties (CPQBA2/39 and PL5) originated from clones of Chinese SI/SII and Vietnamese FI/FII seeds adapted in 1991 at CPQBA experimental field, which were provided by MEDIPLANT (Centre de Recherches SUR LES PLANTES medicinal ET AROMATIQUES - Switzerland) responsible for the work of improvement of seeds. The work in Brazil was accomplished by Dr. Pedro Melillo Magalhães and Dr. Glyn Mara Figueira.

This material was allowed to dry under air circulation (40°C) and ground for use. The resulting powder (2000 g) was submitted to dynamics maceration with ethanol during 2 hours (this procedure was repeated three times) [8].

A 10% lead acetate solution (1 L) was added to the crude ethanolic extract (108 g) dissolved in ethanol (100 mL). This mixture was allowed to stand at room temperature overnight and filtered. The filtrate was extracted with chloroform (3 × 350 mL), dried over MgSO_4_, filtered and dried under vacuum affording the sesquiterpene lactone enriched fraction (Lac-FR) (10 g - 9.3% yield) [8, 12]. This fraction (10 g) was purified on successive column chromatography using silica gel (Merck 7734) (5 × 60 cm) with hexane/ethyl acetate (99:1), R_f_ dihydro-epideoxyarteannuin B: 0.65 between 500–950 mL, [α_D_^20^]: + 49.96° (*c* 0.025 g/mL, CHCl_3_); hexane/ethyl acetate (99:2), R_f_ deoxyartemisinin: 0.61 between 1000–1700 mL, [hexane/ethyl acetate (99:3), [α_D_^20^]: -149.8° (*c* 0.045 g/mL, CHCl_3_); R_f_ artemisinin: 0.57 between 1750–2550 mL, [α_D_^20^]: + 80.93° (*c* 0.025 g/mL, CHCl_3_).

Fractions were monitored by thin layer chromatography, eluent hexane/dichloromethane/methanol (20:79:1), detection anisaldehyde reagent. The physical and spectral data (mass, ^1^H-NMR, ^13^C-NMR) of compounds isolated were consistent with those of artemisinin, deoxyartemisinin and dihydro-epideoxyarteannuin-B reported previously [8].

#### Chromatographic analysis

Development and validation of analytical method by HPLC-IR for evaluation of artemisinin in *A. annua* L. was accordingly to method previously developed and validated by our research group [10]. The method was based on high performance liquid chromatography, using a CN column with mobile phase composed of methanol:H_2_O 50:50 (V/V). The results showed that the method presented linearity from 50 to 1500 μg/ml.

### Pharmacology

#### Drugs

All samples were diluted in a vehicle made of 1% Tween^80^ (Sigma-Aldrich, U.S.A.) in 0.9% saline solution (NaCl diluted in distilled water), considering each different chemical characteristics (hydrophilic/lipophilic). The drugs pentobarbital (Cristália-Brazil), dexamethasone, carrageenan (Sigma-Aldrich, U.S.A.), morphine hydrochloride (Johnson & Johnson, Brazil), indomethacin (Sigma-Aldrich, U.S.A.), naloxone hydrochloride (Sigma-Aldrich, U.S.A.), and the reagents acetic acid and formaldehyde from Sigma-Aldrich, U.S.A. - were used.

#### Animals

Male *Swiss* mice 25–35 g and male Wistar rats 150–250 g body weights were kept at 25 ± 2°C in 12 h light–dark cycles (light phase started at 7:00 am) maintained (10 and 5 animals per cage, respectively) with water and food *ad libitum*, for at least 7 days prior to assays. Separate groups of animals were used for each test, and they were used only once in the experiments. Studies were carried out in accordance with the Current Guidelines for the Veterinary Care of Laboratory Animals - Joint Working Group on Veterinary Care, Finland [13] and were performed under the consent and surveillance of the Institute of Biology Ethics Committee for Animal Research (protocol 3016–1), University of Campinas - Brazil. The number of animals and the intensity of the noxious stimuli were the minimum necessary to obtain reliable data.

### Evaluation of acute toxicity

Groups of male Swiss mice (n = 3) and male Wistar rats (n = 3) intraperitoneally treated with Lac-FR in 30, 75, 100, 300 and 500 mg/kg of a single dose. After administration animals were observed during the first four hours and daily during 14 days. On the 15^th^ day all animals were euthanized by cervical dislocation, followed by necropsy and macroscopic observation of the organs (OECD, [13]; Botham, [14]).

### Evaluation of locomotor activity

The ambulatory behavior was assessed in an open-field as described previously [15, 16]. The apparatus consisted of a plastic box measuring 45 × 45 × 20 cm, with the floor divided into 9 equal squares (15 × 15 cm). The number of squares crossed with all paws (crossing) was counted in a 6-min session for each animal group (n = 6) and considered indicative for the quantification of the locomotor activity. For this purpose, mice were treated intraperitoneally (i.p.) with the Lac-FR (75, 100 and 300 mg/kg), pentobarbital (35 mg/kg), or vehicle (10 ml/kg) 30 min beforehand.

### Writhing test

Groups of *Swiss* mice (n = 6) were treated with vehicle (10 ml/kg), or sesquiterpene lactones fraction (Lac-FR) using 100 and 300 mg/kg doses under intraperitoneal (i.p.) and oral (p.o.) routes. Writhings were induced by an i.p. injection of 0.8% acetic acid solution (10 ml/kg), 30-min after i.p. treatments and 1 hour after p.o. treatments. After this procedure, the numbers of writhings (abdominal constrictions) were cumulatively counted over 15 min, for nociception evaluation [14, 15]. Data represent the average of the total writhing observed per dose administrated.

### Formalin test

The formalin test was carried out as described by Woolfe and Macdonald (1944) [17] with few changes in the protocol [15]. Formalin-induced pain behavior is biphasic: the initial acute phase (0 - 5 min, neurogenic pain) is followed by a relatively short quiescent period, which is then followed by a prolonged tonic response (25 - 40 min, inflammatory pain). Groups of mice (n = 6) were treated i.p. with: vehicle (10 ml/kg, negative control), the positive control for the neurogenic phase morphine (10 mg/kg, determined previously), the positive control for the inflammatory phase indomethacin (30 mg/kg, determined previously), and the dose–response of Lac-FR (30, 100 and 300 mg/kg (i.p.). After 30 min, animals were injected with 20 μl of a formalin solution (formaldehyde 1.2%, in PBS) into the plantar surface of the right hind paw. The total time spent by animal licking or biting the injected paw, an index of nociception, was recorded for the following 40 min.

### Mechanical allodynia induced by Complete Freund’s Adjuvant (CFA)

The procedures were developed and standardized in our laboratory based on the method previously described by Villeti et al. (2003) [18] with changes in protocol and data analysis [19]. Different groups of rats, Wistar male (n = 6), were used during the whole experiment and inflammation was induced with a solution of CFA (1 mg/ml of heat killed *Mycobacterium tuberculosis* in 85% paraffin oil and 15% mannide monooleate) injected (0.1 ml) into the plantar surface of the right hind paw. The left hind paw received the same volume of saline solution (NaCl 0.9% diluted in distilled water) in order to equalize the sensibility of the animals caused by the injection. Mechanical allodynia was assessed using the Dynamic Plantar Anesthesiometer apparatus (Ugo Basile, mod 37450, Italy) which consisted of an elevated wire mesh platform to allow access to the ventral surface of the hind paws. A steel rod (diameter 0.5 mm) was pushed against the hind paw with ascending force (touch stimulator). The force ranged from 0 to 35 g over a 20-s period. When the animal withdrew the hind paw, the mechanical stimulus was automatically stopped, and the force applied by the animal to withdraw the paw was recorded to the nearest 0.1 g. An allodynia score was determined after four consecutive measurements using the touch stimulator sequentially on the left and right hind paw and calculated considering the formula below:


The basal score was measured before CFA injection on day 0, and the animals considered for testing were those with a mean value nearest to 1 (demonstrating no significant difference between both paw stimuli). After CFA injection, measurements were carried out considering two different phases, as follows: 4 h on day 0 (acute pain) and 24 h (sub-acute pain). Vehicle (10 ml/kg), Lac-FR were administered (30, 100 and 300 mg/kg, i.p.) 30-min prior to touch stimulation, in order to evaluate the possible anti-allodynic activity observed for each phase.

### Carrageenan-induced mice paw edema

The anti-inflammatory properties were investigated by using the carrageenan-induced edema model. The procedures used for this study were similar to those described previously with some changes in the protocol and data analysis [20, 21]. Different groups of Swiss mice (n = 6) were treated with the negative control (vehicle), dexamethasone (1 mg/kg, i.p.-positive control) and Lac-FR (100 and 300 mg/kg, i.p.) 2 h before administration of carrageenan (25 μL of a 3% solution carrageenan) injected subcutaneously into the plantar region of the left hind paw. Paw measurements (Plethysmometer 7140 Ugo Basile) were taken at 2, 4, 6 and 24 hours. Results were expressed as variations in time of the paw edema (mL).

### Tail flick

Antinociception was determined using a tail-flick test as described previously with few modifications on the protocol [22, 23]. In brief, the distal one third of the tail was immersed in a water bath maintained at 48 ± 0.5°C. Latency times until a tail-flick response (tail’s abrupt withdrawal) were recorded before (baseline) and at different time points (0.5, 1, 2, 4 and 24 hours) after drug treatment. Separate groups of six mice were treated with morphine (5 mg/kg, i.p.), Lac-FR (30, 100, and 300 mg/kg, i.p.), or an equal volume of vehicle (10 ml/kg). Aiming to evaluate the possible involvement of the opioid system, in another set of experiments (time points 0.5, 1, 1.5, 2, and 4 hours) groups of six mice were treated with morphine (5 mg/kg, i.p.), Lac-FR (100 mg/kg, i.p.), or an equal volume of vehicle, in the presence and absence of the opioid antagonist naloxone (5 mg/kg, s.c.). The antinociception response was presented as percent maximal possible effect (%MPE) as defined by percent maximal possible effect = 100% × (drug response time – basal response time)/(cut-off time – basal response time). A cut-off time of 15 s was applied to avoid tissue damage.

### Statistical analysis

All results were submitted to one way analysis of variance (ANOVA) with repeated measurements, *p ≤ 0.05* was considered the critical level for evaluating significant difference between the control and treated groups, followed by Tukey’s test (one-way ANOVA) or Bonferroni’s test (two-way ANOVA). Prisma® software was used to produce graphs.

## Results

### Phytochemistry

A total 9.3% yield of sesquiterpene lactone fraction (Lac-FR) resulted from the *A. annua* ethanol crude extract with 1.72% artemisinin and 0.31% deoxiartemisinin content determined by HPLC-IR.

### Acute toxicity assessment

The Evaluation of Acute Toxicity determined the doses to be used and the number of animals per group. The administration of 30, 75, 100, 300 and 500 mg/kg doses intraperitoneally, produced no mortality or behavioral disorders in animals after 14-days observation. Lethargy and ptosis, was observed for the 500 mg/kg i.p. dose up to 1 h. Therefore that dose was not considered.

### Evaluation of locomotor activity

**Figure 1:**
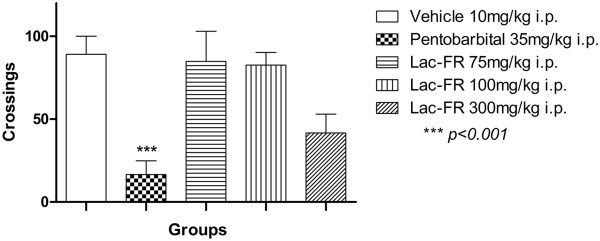
**Evaluation of the locomotor activity of Lac-FR.** Number of crossings in the open-field test of animals treated with vehicle (10 ml/kg, i.p.), pentobarbital (35 mg/kg, i.p.), and Lac-FR (75, 100, and 300 mg/kg) (i.p.). Results expressed as mean ± S.E.M. of 6 animals for experimental groups. (ANOVA and Tukey’s test. ***p < 0.01).

Based on the acute toxicity evaluation, the ranges of dosages tested in the open-field test were 75, 100, and 300 mg/kg (i.p.). Therefore, the dose–response curve designed for this experiment demonstrated that (Figure 1) the negative control group receiving only vehicle (10 mg/kg, i.p.) produced 89.17 ± 10.91 crossings, and the positive control with pentobarbital (35 mg/kg, i.p.) reduced to 16.50 ± 8.31 crossings (81.48% reduction). The i.p. treatments with the fraction Lac-FR demonstrated 84.83 ± 18.26 (75 mg/kg), 82.67 ± 7.62 (100 mg/kg), and 41.67 ± 11.33 (300 mg/kg) crossings. The reduction of crossings by 53.25% observed for the 300 mg/kg group, compared to the negative control, indicated that for the next antinociceptive experiments, this dosage may not indicate only antinociception, but also cognitive and/or muscular alterations. These results allowed us to determine 100 mg/kg as the main dosage to be considered in the antinociceptive properties of the Lac-FR.

### Writhing test

**Figure 2:**
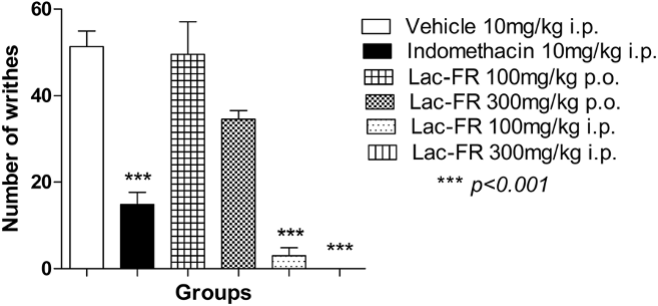
**The antinociceptive activity of Lac-FR in the writhing test.** Abdominal constrictions induced by acetic acid (0.8% in saline) i.p. in mice previously treated (30 min) with control vehicle (10 ml/kg i.p.), indomethacin-positive control (10 mg/kg i.p.) or Lac-FR (100 and 300 mg/kg) (i.p. and p.o.). Results expressed as mean ± S.E.M. of 6 animals per experimental groups (ANOVA and Tukey’s test. ***p < 0.001).

The non-specific writhing test was employed to explore the potential use of Lac-FR for pain releif when tested with two different routes: oral (p.o.) and intraperitoneal (i.p.). The Figure 2 demonstrated that the negative control group vehicle (10 ml/kg i.p.) produced 48.17 ± 5.21 writhings whereas the positive control indomethacin (10 mg/kg i.p.) showed a reduction of 69.3% by 14.80 ± 2.81 writhings (*p < 0.001*). Lac-FR samples were then administrated by both p.o. and i.p. for kinectis comparisons. The treatments of Lac-FR with 100 and 300 mg/kg p.o. doses demonstrated writhings around 49.50 ± 7.5 and 34.60 ± 1.9, respectively. For the i.p. administration with the same 100 and 300 mg/kg doses, the number of writhings decreased to 3.0 ± 1.89 (93.7%) and zero (100%), respectively (*p < 0.001*). These results showed that the oral treatment with Lac- did not decrease significantly the abdominal constrictions compared to the vehicle group, so that the oral treatment was excluded of the next evaluations of the aninociceptive properties.

### Formalin test

**Figure 3:**
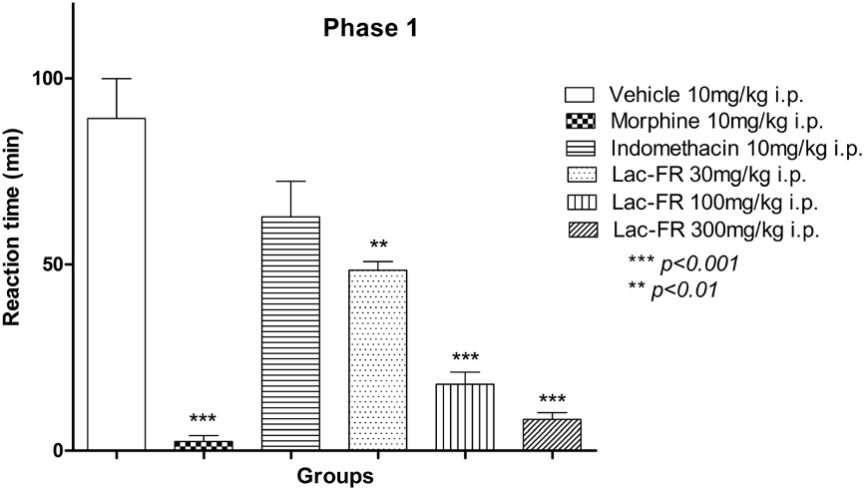
**Antinociceptive activity of Lac-FR related to chemical neurogenic pain stimulus.** First phase (0–5 min) for reactivity time to the intraplantar application of formalin (formaldehyde 1.2% in PBS) in the hind paw of mice previously treated i.p. (30 min) with control vehicle (10 ml/kg i.p.) morphine (10 mg/kg i.p.) indomethacin (10 mg/kg i.p.) or fraction (30, 100, and 300 mg/kg i.p.). Results expressed as mean ± S.E.M. of 6 animals for experimental groups (ANOVA and Tukey’s test. ***p < 0.001, **p < 0.01).

We next explored how effective the fraction Lac-FR could be against neurogenic and inflammatory pain stimuli under the formalin test. Results presented in the first phase (Figure 3) demonstrating the neurogenic response (0-5 min), showed that the negative control group (vehicle 10 ml/kg, i.p.) produced a reaction time of 89.17 ± 10.74 seconds. The positive controls morphine (10 mg/kg, i.p.) 2.50 ± 1.58 seconds (97% reduction; *p < 0.001*), and indomethacin (10 mg/kg, i.p.) 62.80 ± 9.47 seconds (no significant reduction). For this first phase, the fraction Lac-FR showed reaction times by 48.40 ± 2.35 (30 mg/kg), 17.80 ± 3.27 (100 mg/kg) and 8.33 ± 1.82 (300 mg/kg) seconds reducing the pain stimuli around 45.72%, 80.03%, and 90.65%, respectively (*p < 0.001*).

**Figure 4:**
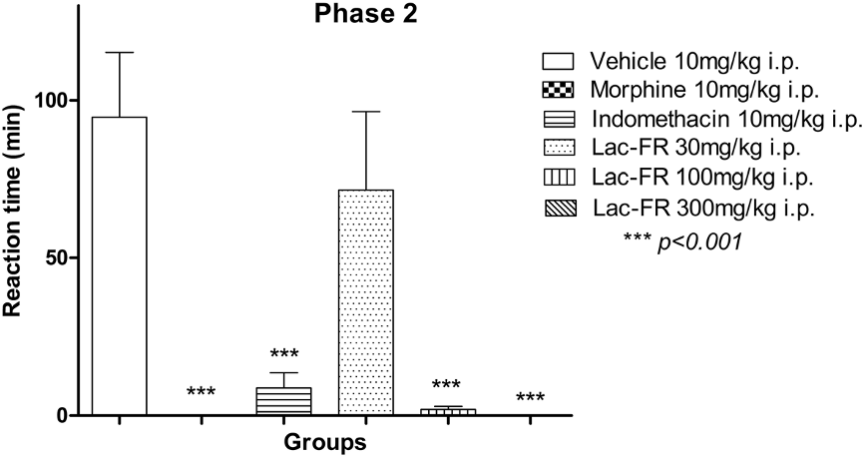
**Antinociceptive activity of Lac-FR related to chemical inflammatory pain stimulus.** Second phase (25–40 min) for reactivity time to the intraplantar application of formalin (formaldehyde 1.2% in PBS) in the hind paw of mice previously treated i.p. (30 min) with control vehicle (10 ml/kg i.p.), morphine (10 mg/kg i.p.), indomethacin (10 mg/kg i.p.), or fraction (30, 100, and 300 mg/kg). Results expressed as mean ± S.E.M. of 6 animals for experimental groups (ANOVA and Tukey’s test. ***p < 0.001).

Moreover, results presented in Figure 4 for the second phase of the formalin test (25–40 min) representing the inflammatory response, the group that received only vehicle (i.p.) showed a reaction time of 94.60 ± 20.56 seconds. The positive control groups morphine (10 mg/kg, i.p.), and indomethacin (10 mg/kg, i.p.), reduced the reaction time by 0 (100%) and 8.66 ± 4.84 seconds (97.8%), respectively (*p < 0.01*). The i.p. treatments with the fraction Lac-FR produced a significant reduction of the reaction times compared to the control group, producing 71.60 ± 24.83 (30 mg/kg), 2.0 ± 0.94 (100 mg/kg), and zero (300 mg/kg) seconds. On this phase, the treatment with 100 mg/kg and 300 mg/kg reduced stimuli by 97.8% and 100%, respectively (*p < 0.01*), and motivated us to further explore the antinociceptive properties of the fraction Lac-FR related to inflammatory stimuli.

### Mechanical allodynia assessment

**Figure 5:**
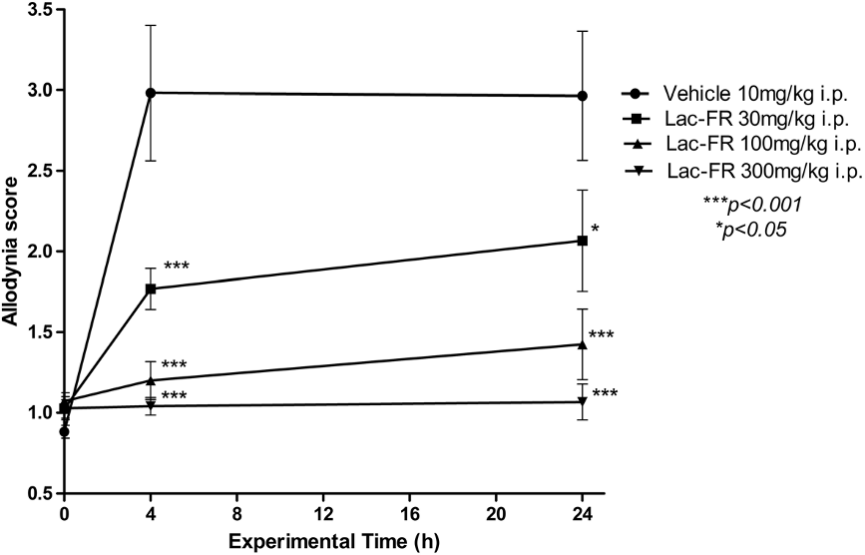
**Determination of the allodynia score of Lac-FR in the CFA model of persistent pain.** Graph demonstrating the decrease on allodynia score related to the touch stimulation exerted on the surface of the left and right hind paws caused by injection of saline solution and CFA (0.1 ml) respectively, producing persistent pain sensitization in rats treated with the Lac-FR (30, 100, and 300 mg/kg, i.p.), compared to the control group (vehicle, 10 ml/kg i.p.). Results expressed as reduction score means ± S.E.M. of 6 animals for experimental group (ANOVA and Bonferroni’s test. ***p < 0.001, *p < 0.05).

Evaluating whether the fraction Lac-FR could be effective in the persistent pain caused by the intraplantar injection of CFA in the mice, the mechanical allodynia showed in the Figure 5 demonstrated the effectiveness of Lac-FR in a dose-dependent manner. Our results showed that Lac-FR produced a significant anti-allodynic activity reducing the allodynia score compared to the control group vehicle (2.9 ± 0.12) during the acute phase (4 h post CFA) of the test. The allodynia scores observed for the fraction Lac-FR at 4 h time were 1.76 ± 0.12 (30 mg/kg), 1.20 ± 0.11 (100 mg/kg), and 1.04 ± 0.05 (300 mg/kg). The reductions of the allodynia score on this phase were 40.93%, 59.73% and 65.10%, respectively (*p < 0.001*). In the sub-chronic phase of the test (24 h post CFA), the control group vehicle showed a 2.8 ± 0.08 allodynia score. The i.p. treatment with the fraction Lac-FR presented allodynia scores by 2.06 ± 0.31 (30 mg/kg), 1.42 ± 0.21 (100 mg/kg), and 1.06 ± 0.11 (300 mg/kg), reducing around 30.4%, 52.02%, and 64.18% compared to the control group. This result highlighted the activity of the fraction Lac-FR in inflammatory pain states, corroborating the phase II of the formalin test.

### Carrageenan-induced paw edema

**Figure 6:**
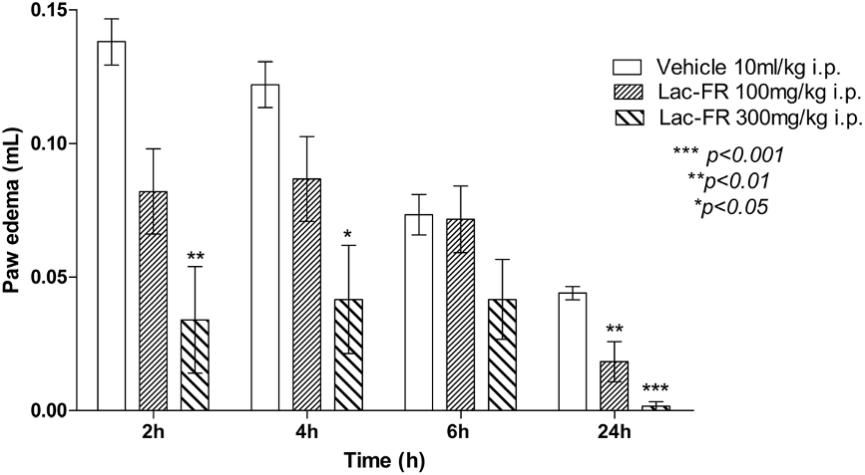
**Evaluation of the potential anti-inflammatory effects of Lac-FR.** Effect of the Lac-FR on carrageenan-induced paw edema expressed as paw edema (mL) per time after inflammation induction. Mice were pretreated i.p. with Lac-FR (100 and 300 mg/kg), and vehicle (10 ml/kg i.p.), 30 min before the intraplantar 2.5% carrageenan injection. Paw edema was evaluated in plethysmometer 2, 4, 6, and 24 hours after inflammation induction. (ANOVA and Bonferroni’s test. ***p < 0.001, **p < 0.01; *p < 0.05).

Aiming to evaluate the influence of the fraction Lac-FR in the inflammatory edematogenic processes, the intraplantar injection of carrageenan producing edema was carried out. Results presented in Figure 6, demonstrated that the fraction Lac-FR was significantly effective on reducing edema compared to the control group within 2 h, 4 h, and 6 h post-carrageenan injection. These reductions were observed after i.p. treatments with Lac-FR 100 and 300 mg/kg doses (*p < 0.001*). The most important conclusion of this test is the effectiveness of the fraction not only in the perception of the inflammatory stimuli, but also decreasing inflammatory mediators causing edema.

### Tail flick test

**Figure 7:**
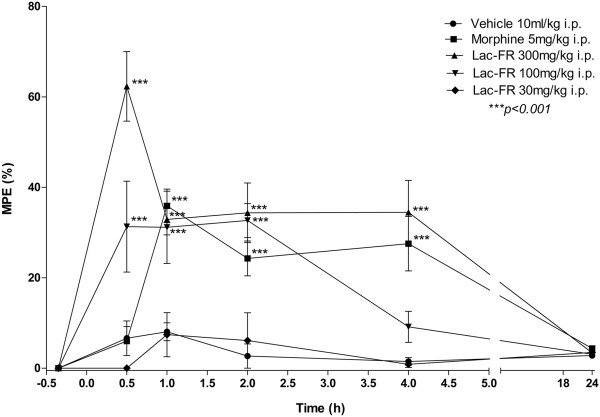
**The antinociceptive effects of Lac-FR against thermal stimulus.** Effect of the Lac-FR on tail flick test. Mice were treated i.p. with Lac-FR (30, 100 and 300 mg/kg i.p.), morphine (5 mg/kg i.p.), and vehicle (10 ml/kg i.p.), 0.5 h before evaluation. Evaluation times were 0.5, 1, 2, 4 and 24 hs. (ANOVA and Bonferroni’s test. ***p < 0.001).

**Figure 8:**
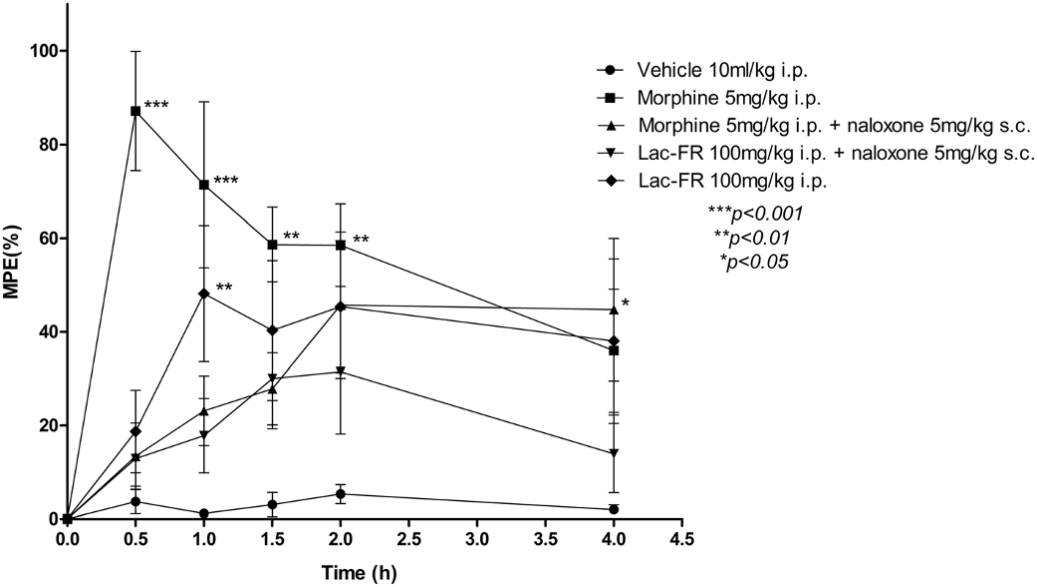
**Evidences of the involvement of opioid system in the antinociception caused by Lac-FR.** The antinociceptive effect increase of morphine (5 mg/kg i.p.), morphine (5 mg/kg i.p.) associated with naloxone (5 mg/kg s.c.), and Lac-FR (100 mg/kg i.p.) associated with naloxone (5 mg/kg s.c.), after one hour period were 70.21% (morphine), 21.94% (morphine with naloxone), 16.62% (Lac-FR with naloxone), and 46.96% (Lac-FR 100 mg/kg i.p.), respectively. Evaluation times were 0.5, 1, 2, 4, and 24 hs. (ANOVA and Bonferroni’s test. ***p < 0.001, **p < 0.01, *p < 0.05).

The first phase of the formalin test, revealing activity under neurogenic stimulus, motivated us to further explore this mediation. For this purpose, the tail-flick was employed. The time-points for the %MPE analysis were 0.5, 1, 2, 4, and 24 h after treatments, and fraction Lac-FR was effective within 4 h of the test. The most important results are the ones observed after 1, 2, and 4 h mainly with the 100 mg/kg (*p < 0.001*), which is a dosage effective under thermal neurogenic stimulus with no influence in the open-field test (Figure 7).The antinociceptive effect on the tail flick was also evaluated using the opiod antagonist naloxone (Figure 8). In this experiment only a 100 mg/kg Lac-FR dose was tested to observe the antagonism effect after the pre-treatment with naloxone. The %MPE increase of morphine (5 mg/kg i.p.), morphine (5 mg/kg i.p.) associated with naloxone (5 mg/kg s.c.), and Lac-FR (100 mg/kg i.p.) associated with naloxone (5 mg/kg s.c.), after one hour period were 70.21% (morphine), 21.94% (morphine with naloxone), 16.62% (Lac-FR with naloxone), and 46.96% (Lac-FR 100 mg/kg i.p.), respectively. With this result, the influence of the pre-treatment with naloxone was observed.

## Discussion

During the last three decades sesquiterpenoid lactones have emerged as one of the largest groups of plant products that have a broad range of biological activities such as anti-inflammatory effect. *Artemisia spp*. characteristically contains sesquiterpene lactones. Significant antiinflammatory effects of some sesquiterpene lactones isolated from Artemisia spp. have been reported [24].

Artemisinin (Qinghaosu), a natural compound identified in *Artemisia annua* L., is an effective drug mainly used against cerebral malaria. The action of this drug is immediate and parasitaemia in the treatment of drug-resistant malaria is rapidly reduced, justifying the industrial production of artemisinin. The worldwide demand for artemisinin derivatives has increased due to the benefits provided when compared to other medications employed to treat malaria. This disease is still one of the major in the world, causing physical and economic problems in tropical regions. Therefore there is a great demand for the production of artemisinin derivatives. Our research group developed an industrial production of this potent antimalarial drug, including strategies for enhancing yield using inexpensive and easy steps lowering the cost three-fold [9]. Nevertheless the large amount of by-product generated in the process prompted studies on the extract’s pharmacological potencies [7, 8].

According to Chadwick et al. (2013) [1] and Chaturvedi (2011) [5], sesquiterpenes lactones, from Asteraceae, may play a highly significant role in human health, both as part of a balanced diet and as pharmaceutical agents. They are described as the active constituents of a variety of medicinal plants used in traditional medicine because of the wide variety of biological and pharmacological activities such as antimicrobial, cytotoxic, antiinflammatory, antiviral, antibacterial, antifungal activities, effects on the central nervous and cardiovascular systems as well as allergenic potency. Previously we reported the antiulcer activity of the extract on ethanol and indomethacin-induced ulcer models [7–9]. Herein we report the antinociceptive and anti-inflammatory activity of Lac-FR evaluated in animal experimental models with mice and rats.

Acute toxicity evaluation was undertaken in animal experimental models using both mice and rats with 30, 75, 100, and 300 mg/kg doses not revealing any toxic effects. Therefore those same doses were considered in the ones uses throughout this study.The antinociceptive activity was evaluated using writhing, formalin, CFA-induced persistent pain, and tail-flick tests. The writhing test determined the most convenient route of administration (100 and 300 mg/kg i.p.), establishing that as route of choice for sample treatment (Lac-FR) (Figure 1).

The open field test (Figure 2) was performed in order to exclude the possibility that the antinociceptive action could be related to non-specific disturbances in the locomotor activity of the animals [25]. That test is generally believed to be a result from brain activation, which is manifested as an excitation of central neurons and an increase in cerebral metabolism [15, 26]. The open field test identified that doses up to 100 mg/kg (i.p.) did not interfere in the locomotors activity (100 mg/kg).

The anti-inflammatory activity of Lac-FR was determined both by the second phase of the formalin test and further by paw edema model induced by carrageenan. The relationship of the central nervous system involved with pain was evaluated by the tail flick model, including evaluation of the opioid system using naloxone as antagonist.

Acetic-acid induced pain (writhing test) is a screening tool for the assessment of analgesic or antiinflammatory properties of natural or synthetic compounds. The acid acts indirectly by inducing the release of endogenous mediators, which stimulate the nociceptive neurons. Inflammatory pain transmission primarily involves peripheral polymodal receptors around small vessels which signal to the CNS via sensory afferent C-fibers entering the dorsal horn. However this chemical method has good sensitivity but poor specificity allowing misinterpretation of the results, because this is an unspecific stimulus for nociception, sensible to drugs with different mechanisms. This problem can be avoided by complementation with other nociception models reported herein [15, 27, 28].

This assay provides a convenient stimulus for screening, because the intensity of response depends on the interaction of several factors, neurotransmitters and neuromodulators that determine nociception, such as kinines, acetylcholine, substance P and prostaglandins. Therefore, this model is responsive to analgesic substances possessing the most varied action mechanisms, being sensitive to drugs with analgesic activity such as aspirin, kinin receptor antagonists (bradykinin, kallidin or T-kinin), and opioid analgesics with central or peripheral action. This model permitted evaluation of antinociceptive activity caused by both neurogenic and/or inflammatory pain [25]. Thus, a positive result with this test does not necessarily mean there is analgesic activity. Nevertheless, because all analgesics inhibit abdominal cramps, this method is useful for sifting molecules whose pharmacodynamics properties are unknown [29].

In order to determine if the antinociceptive activity was through neurogenic or inflammatory stimulus the formalin test was employed. Neurogenic pain is caused by the direct activation of nociceptive nerve terminals, whereas inflammatory pain is mediated by a combination of peripheral input and spinal cord sensitization. Formalin produces significant increases in spinal levels of different mediators related to both neurogenic (amino acids, kinins) and inflammatory pathways (prostaglandins, leukotrienes) [15, 16].Results presented herein present consistent data to support antinociceptive activity in both the acute phase reflecting direct activation of nociceptors and late phase reflecting inflammation. In the first 5 minutes (phase 1) a significant decreased was observed for 100 and 300 mg/kg Lac-FR compared to morphine (10 mg/kg, i.p.) positive control (Figure 3). These findings suggest that the activity of the sample under test might have a relationship with desensitization of receptor sensitive to chemical stimulus. The second phase also demonstrated a significant reduction of the reaction time suggesting that Lac-FR has a mechanism of action that act through both neurogenic and anti-inflammatory mediators.

To better understand the inflammatory pain pathway the CFA-induced persistent pain model was evaluated. Persistent pain caused by CFA intraplantar injection involves central sensitization due to the release of multiple inflammatory and pain mediators that account for sensitivity increase of both peripheral sensory afferents at the injury site, and in the central nervous system [19, 30]. The arrival of sensorial information from nociceptors into the dorsal horn considerably alters the level of activity within the cord as both excitatory and inhibitory systems can impinge upon spinal neuronal activity. This forms the basis of central hypersensitivity which results in increased responsiveness of dorsal horn neurons, often observed in persistent inflammatory and neuropathic states of pain [19, 31].The three Lac-FR doses evaluated (30, 100, and 300 mg/kg i.p.) decreased the mechanical allodynic stimulus. The two higher doses showed significant activity both in the acute (4 h after CFA injection) and sub-acute phase (24 h after CFA injection) (Figure 5).

The intraplantar injection of carrageenan induces both hipernociception and edema formation [32]. This inflammatory method has become a widely used model for studying acute inflammation [20]. In this model, carrageenan evokes a very inflammatory and nociceptive response characteristic, which is mediated by different groups of endogenous substances that stimulate chemo sensitive nociceptors, thus playing a major role in the development of inflammatory pain [19, 33, 34]. Sample Lac-FR with 100 and 300 mg/kg (i.p.) showed decreased paw edema after 2 h time suggesting a relationship with inflammatory mediators such as prostaglandins, leukotrienes among others (Figure 6).Considering that sample Lac-FR demonstrated activity in phase 1 of formalin test, that demonstrated a relationship with neurogenic pain, the tail flick model was employed to verify if the sample had the capacity to reduce thermal stimulus. Both the samples (100 and 300 mg/kg i.p.) and morphine (5 mg/kg i.p.) demonstrated the same effect after elapse of an hour. Thereafter Lac-FR (300 mg/kg i.p.) and positive control (morphine) maintained the same antinociception index up to four hours of experimental time. These finding suggest that the sample has an antinociceptive pathway with both peripheral characteristics and central nervous system component (Figure 7). Moreover employing the same assay the possible involvement of opioid mechanism was evaluated injecting naloxone, an opioid antagonist reverted the analgesic activity.

The differences in activity among individual sesquiterpene lactones may be explained by differences in the number of alkylating elements, lipophilicity, molecular geometry, and the chemical environment of the target sulfhydryl group. Although, the exact mechanism of action of sesquiterpene lactones are not well known, they have been documented through the various published reports on the biological activity displayed by the majority of sesquiterpene lactones with α*-*methylene-γ*-*lactones and α,β-unsaturated cyclopentenone ring [5].

The presence of such groups in sesquiterpene lactones has been significant in eliciting potent inhibitory activity against iNOS-dependent [11]. The most widely published class of natural products cited as inhibitors of NF-ƙB (nuclear factor kappa B) are the sesquiterpene. NF-ƙB is a protein that mediates immune response in humans by controlling response of other effectors such as cytokines, inflammatory molecules and cell adhesion molecules [1, 5]. The current understanding of the NF-ƙB cascade provides the biochemist and natural product scientist with a tantalising opportunity of potential targets [11]. Other theories on the anti-inflammatory effects of sesquiterpene lactones include activation of p53 and an increase in ROS as cytotoxic effects of sesquiterpene lactones [1].

The results described herein are the first report of the antinoniceptive and antiinflammatorry activity of Lac-FR obtained from *A. annua* extract with 1.72% artemisinin and 0.31% deoxiartemisinin content.

## Conclusions

The most relevant findings of the present work were that Lac-FR obtained from *Artemisia annua* L. extract have potential to be used for pain relief revealed by chemical-induced nociception assays in mice; the i.p. treatment produced a significantly antinociceptive effect in the writhing test and produced a relevant reduction in the reaction time of the animals in both phases of the formalin test. Also, the i.p. treatment significantly reduced the sensitivity to mechanical allodynia stimulus and reduced the paw edema caused by carrageenan injection. Finally the i.p treatment promoted high antinociceptive activity in tail flick model suggesting relationship with the opioid system. Further studies are being undertaken to elucidate the active components involved with the antinociceptive activity as well as evaluate synergy effect that is seen by combination of substances that is greater than would be expected from consideration of individual contributions.
